# Optimizing gene set annotations combining GO structure and gene expression data

**DOI:** 10.1186/s12918-018-0659-6

**Published:** 2018-12-31

**Authors:** Dong Wang, Jie Li, Rui Liu, Yadong Wang

**Affiliations:** School of Computer Science and Technology, Harbin Institute of Technology, West Da-Zhi Street, Harbin, China

**Keywords:** Gene ontology, Gene set enrichment, Clustering algorithm, Gene set annotation

## Abstract

**Background:**

With the rapid accumulation of genomic data, it has become a challenge issue to annotate and interpret these data. As a representative, Gene set enrichment analysis has been widely used to interpret large molecular datasets generated by biological experiments. The result of gene set enrichment analysis heavily relies on the quality and integrity of gene set annotations. Although several methods were developed to annotate gene sets, there is still a lack of high quality annotation methods. Here, we propose a novel method to improve the annotation accuracy through combining the GO structure and gene expression data.

**Results:**

We propose a novel approach for optimizing gene set annotations to get more accurate annotation results. The proposed method filters the inconsistent annotations using GO structure information and probabilistic gene set clusters calculated by a range of cluster sizes over multiple bootstrap resampled datasets. The proposed method is employed to analyze p53 cell lines, colon cancer and breast cancer gene expression data. The experimental results show that the proposed method can filter a number of annotations unrelated to experimental data and increase gene set enrichment power and decrease the inconsistent of annotations.

**Conclusions:**

A novel gene set annotation optimization approach is proposed to improve the quality of gene annotations. Experimental results indicate that the proposed method effectively improves gene set annotation quality based on the GO structure and gene expression data.

## Background

**Fig. 1:**
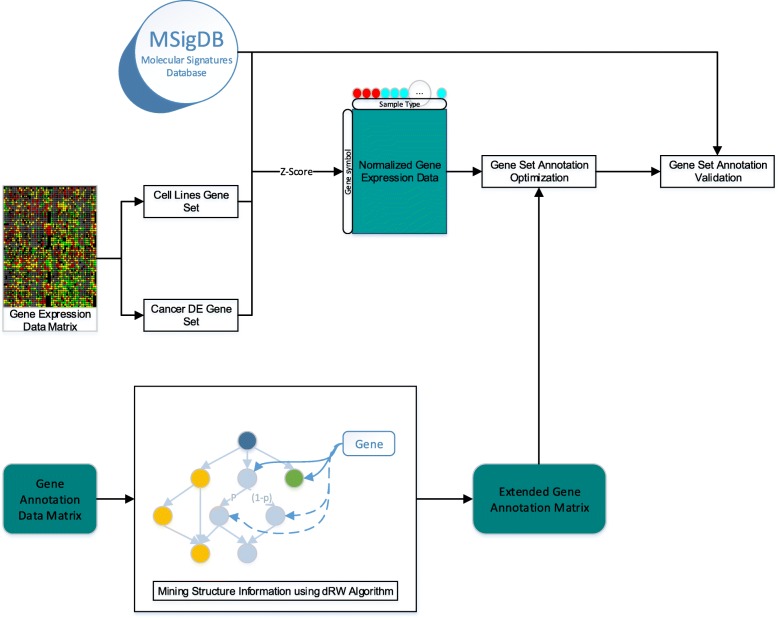
Overview of the proposed method. The cell line and cancer gene expression are introduced into our experiment. All of gene expression data are normalized us by Z-score. Differentially expressed(DE) genes are introduced as the genes to be optimized. Gene annotation data obtain the structure information using the dRW algorithm. Then, the normalized gene expression data and gene annotations contained the GO structure information will be introduced into the gene set annotation optimization algorithm. Gene set annotation optimization: gene sets divided by the clustering algorithm will own similar annotations using gene expression data. Finally, the proposed method and the state-of-the-art methods are evaluated using three metrics

With the development of next-generation sequencing technology, a large amount of genomic data generated in biological and medical fields. It has become an important task how to interpret these data and make full use of these data to help researchers understand the mechanism of complex diseases. Currently, some gene knowledge databases and annotation tools, such as Gene Set Enrichment Analysis (GSEA) [1, 2], Kyoto Encyclopedia of Genes and Genomes (KEGG) [3] and Gene Ontology (GO) [4] have been developed to help researchers annotate and understand gene functions. For example, KEGG is a knowledge base for systematic analysis of gene functions, linking genomic information with higher order functional information. GO is widely used to annotate and analyze gene sets from complex diseases. However, the annotation results from GO are incomplete and overly general [5] and manual annotations are time-consuming and laborious. Thus, some computational methods are developed to address these issues. Masseroli et al. [6] proposed a modified Probabilistic Latent Semantic Analysis (pLSA) method based on credible GO annotations to predict the unknown functions of genes. Compare with SVD method [7] in different types of data, the pLSA has better performance. In addition to pLSA, Frasca et al. [8, 9] developed a neural-network-based imbalance-aware algorithm called COSNet to predict the unknown functions of genes. The COSNet algorithm is compared with many methods, however, its accuracy is not very high. Yu et al. [10] proposed downward random walk (dRW) method on a gene ontology to predict gene product functions. Compared with COSNet and pLSA, the dRW algorithm has better performance and it is mainly based on gene ontology structure to expand the annotations, which is in line with our requirements. In the paper, it is introduced into our algorithm to construct annotation matrix. Some other methods are also proposed to improve the quality of gene set annotations. Huang et al. [11, 12] established the DAVID database, which allows users to annotate gene sets and analyze gene set functions for different species, but not different diseases. Faria et al. [13] proposed a method based on association rule mining to identify the relationship between different GO terms. QuickGO [14] was developed to construct GO slims according to the user’s needs and filtered some annotations to some specific GO terms. These databases and methods are mainly based on the relationship of GO terms or related literatures to reconstruct gene annotations. However, gene expression data are not combined with existing annotation data to improve the quality of gene set annotations and increase specificity of gene set annotations. In order to improve the quality of gene annotations and enable more accurate and reproducible gene set annotation analysis. Frost et al. [15] proposed a method for optimizing gene set annotations via entropy minimization over variable clusters (EMVC), which filters inconsistent annotations for gene sets by mRNA expression levels measured using RNA-seq or microarray technology. The EMVC algorithm can remove unreliable annotations and make an improvement on the enrichment power and replication. Although EMVC optimizes gene annotations, it only considers the structural information of gene expression data. The GO structure information and probabilistic gene clustering results are not introduced to improve the quality of annotations. The correlation between gene set annotation results and gene expression data need to further improve. In the paper, we incorporated GO structure information and gene expression data to optimize the gene set annotations. The overview of the proposed algorithm is shown in Fig. 1.

## Methods

The proposed method optimizes gene set annotations using gene expression data and structure information of gene ontology. The process of this algorithm is as follows:

(i) In the process of data preprocessing, gene expression data will be normalized by Z-score. For cancer gene expression data, the differentially expressed genes will be selected for the follow analysis.

(ii) The extension of gene (gene product) annotations is quantified by integrating the random walks with restart algorithm [16] and the true path rule [17] and the extended annotation matrix will contain GO structure information.

(iii) In the process of gene annotation optimization, Genes are divided into several categories created by the clustering algorithm using gene expression data. Then a cluster contained the largest number of annotations for each GO term will be retained as a result of the gene annotations. Different from the previous methods, we convert the clustering results into the probability values and use it to optimize the annotation matrix. After optimizing by the GO structure and gene expression data, genes in the same cluster (gene set) in the annotation matrix will be given similar annotations, namely, the optimization algorithm successfully improves the quality of gene annotations.

### Annotation matrix construction

The classical annotation matrix is generally a binary matrix and it places all annotations on the same horizontal line, e.g. all the gene annotation information is converted into 0 or 1 in the annotation matrix [15]. In the paper, the elements of annotation matrix are probability values which more fully reflects the annotation information based on the GO structure from downward random walk algorithm [10] as shown Eq. (1).

1 $$ {\begin{aligned} A &= \left[ \begin{array}{ccc} a_{1,1} & \cdots & a_{1,n}\\ \vdots & \ddots & \vdots\\ a_{t,1} & \cdots & a_{t,n} \end{array} \right ] \\ a_{i,j}&= \left\{ \begin{array}{ll} 1\ \ &gene\ j\ is\ annotated\ by\ term\ i\ \\ &in\ the\ existing\ GO\ data\\ p(i,j) & the\ probability\ of\ \\ & unknown\ annotation\ \\ & for\ gene\ j\ and\ term\ i \end{array}\right. \end{aligned}}  $$

where *a*_*i*,*j*_ denotes gene *j* is annotated by term *i*. The *p*(*i*,*j*) denotes the predicted probability that gene *j* is annotated by term *i* using downward random walks algorithm for the unknown annotation for gene *j* and term *i*. (*i*=1,⋯,*t*, *j*=1,⋯,*n*).

The dRW algorithm has been used to obtain the GO structure information in this article. The first step in the construction of annotation matrix is to quantify the similarity between different GO terms. In this article, we used Gene Ontology semantic similarity Tool (GOssTo) [18] to calculate semantic similarity and selected Lin’s [19] similarity measuring method.

After calculating the semantic similarity, downward random walks with restart [16] on the GO directed acyclic graph (DAG) has been introduced to find out unknown gene annotations. GO annotations abide by the true path rule, namely, if a gene is annotated with a GO term, it will be annotated with all of the ancestor terms of this specific term in the GO DAG. Thus gene annotation extension is mainly how to establish links between genes and the offspring of existing annotated terms. Downward random walks with restart algorithm can chase down the potential functions of a gene for the available terms associated to the same gene.

The adjacency matrix of GO DAG is constructed to show the association of terms. It is defined as follows:

2 $$ \begin{aligned} X &= \left[ \begin{array}{ccc} x_{1,1} & \cdots & x_{1,t}\\ \vdots & \ddots & \vdots\\ x_{t,1} & \cdots & x_{t,t} \end{array} \right]\\ x_{p,q} &= \left\{ \begin{array}{ll} 1\ & term\ q\ is\ child\ of\ term\ p\\ 0 & otherwise \end{array}\right. \end{aligned}  $$

where *p*=1,⋯,*t*, *q*=1,⋯,*t*.

After getting the adjacency matrix *X*, the corresponding probability matrix *T* is constructed using the semantic similarity matrix *SSM* and adjacency matrix *X*. For term *p* and term *q*, we use *trans*(*p*,*q*)=*SSM*(*p*,*q*)×*X*(*p*,*q*) to filter the semantic similarity between different terms and represent the random walk probability between different terms. Since we need to get the probability values, we normalize the corresponding probability matrix *T* by the following formula:

3 $$ T(p,q)=\frac{trans(p,q)}{\sum_{m\in ancestor(term q)} trans(m,q)}  $$

where *ancestor*(*termq*) denotes all ancestor terms ofterm *q*.

The downward random walkers iteratively reach the descendant nodes of the starting term according to the corresponding probability matrix *T*. The final iteration process converges to the steady state. The process is defined as follows:

4 $$ R^{iter+1}=\beta R^{iter} T + (1-\beta)I  $$

where *β*∈[0,1] is the restart probability between different terms, 1−*β* is the probability of a random walker staying the current term. *I* is an identity matrix. *R*^*iter*^ represents the current transition matrix and *R*^*iter*+1^ represents the transition matrix at the next moment. When the difference between *R*^*iter*+1^ and *R*^*iter*^ is less than a fixed value, the transition matrix *R* reaches a steady state, and at this point, the iterative process is terminated, and *R*^∗^ is obtained. Here the difference between *R*^*iter*+1^ and *R*^*iter*^ will be calculated using the following formula:

5 $$ \sum_{p=1}^{t}\sum_{q=1}^{t}\left|R_{p,q}^{iter+1}-R_{p,q}^{iter}\right|<\epsilon  $$

where *t* denotes the number of terms. The *ε* denotes a preset value. When the value on the left side of the inequality is less than *ε*, the iterative process will be terminated.(according to our empirical study, the number of iteration is less than 15) And the transition matrix at this time is said to be in a steady state. (*p*=1,⋯,*t*, *q*=1,⋯,*t*)

The process of downward random walk algorithm will achieve the steady state after several iterations. Finally, the transition probability matrix in the steady state will be combined to construct gene annotation probability matrix. It is defined as follows:

6 $$ A(i,j) = \sum_{e \in \chi_{j}}{R^{*}(e,i)} \ \ \ \ s.t.\ R^{*}(e,i)>\theta  $$

where *θ* is the row mean of the transition probability matrix in the steady state. Its main purpose is to remove those very small probability values. *χ*_*j*_ represents the set of GO terms annotated to the gene *j*. The term e represents the terms that do not annotate the gene *j*. *A*(*i*,*j*) is the probability that the gene *j* is annotated by the term *i*. *R*^∗^ is the transition probability matrix in the steady state.

Finally, we can get the gene annotation matrix contained the GO structure information. Compared to the traditional method, threshold is not directly introduced to decide whether to annotate genes using GO terms in this article. Probability values are retained as inputs to the gene annotation optimization algorithm to compensate for the inability of the optimization algorithm to increase annotation data.

### Gene annotations optimization algorithm

In this section, we introduce the process of gene set annotation optimization algorithm. Our main goal is to annotate the same function for genes with similar expression patterns based on the gene expression data, and filter out inconsistent functional annotation. The algorithm is designed as follows:

- Genes are divided into *k* partitional clusters through clustering algorithm (e.g. K-means) based on gene expression data and establish a category matrix C according to cluster result.
  7 $$  C= \left[ \begin{array}{ccc} c_{1,1} & \cdots & c_{1,k}\\ \vdots & \ddots & \vdots\\ c_{n,1} & \cdots & c_{n,k} \end{array} \right ] \ c_{j,l}= \left\{ \begin{array}{ll} 1\ \ &gene\ j\ belongs\ to\ cluster\ l\\ 0 & otherwise \end{array}\right.  $$
  where *c*_*j*,*l*_ denotes gene *j* belongs to cluster *l*. (*j*=1,⋯,*n*, *l*=1,⋯,*k*)
- Let *A*×*C*, get annotation statistic matrix *S*.
  $$S= \left[ \begin{array}{ccc} s_{1,1} & \cdots & s_{1,k}\\ \vdots & \ddots & \vdots\\ s_{t,1} & \cdots & s_{t,k} \end{array} \right] $$
- Normalize the annotation statistic matrix *S* by Eq. (8)
  8 $$ s_{i,j}=\frac{s_{i,j}}{sum(s_{i,*})}  $$
  where *sum*(*s*_*i*,∗_) is the sum of row *i*.
- Calculate the optimized annotation matrix *A*^∗^
  9 $$ A^{*}= \left[ \begin{array}{ccc} a_{1,1}^{*} & \cdots & a_{1,n}^{*}\\ \vdots & \ddots & \vdots\\ a_{t,1}^{*} & \cdots & a_{t,n}^{*} \end{array} \right] a_{i,j}^{*}=a_{i,j}c_{j,kmax}s_{i,kmax}  $$
  where *kmax* is the column with the max probability of each row in the matrix *S*. Each row of *S* will be used to find the largest subset of annotations. If there are multiple clusters with same values, the algorithm will select one cluster randomly.
- Through *Z*−1 iterations, get *Z*−1 optimized annotation matrix $A_{k}^{*}$ (*k*=2,⋯,*Z*) and the average optimized annotation matrix.
  10 $$ A_{ave}^{*}=\frac{1}{Z-1}\sum_{k=2}^{z} A_{k}^{*}  $$
- Calculate the final optimized annotation matrix $A_{final}^{*}$ after *N* bootstrap resample datasets
  11 $$ A_{final}^{*}=\frac{1}{N}\sum_{k=2}^{z} A_{ave}^{*}  $$

## Results

### Experimental evaluation

In this section, the proposed method was evaluated using gene expression data (p53 cell lines, colon cancer and breast cancer) and the Molecular Signatures Database (MSigDB) [20, 21] gene sets which are widely used [1, 15, 22–25]. MSigDB includes 522 C2 gene sets and 431 C4 cancer modules. The p53 gene expression data [26] are from MSigDB repository. The colon and breast cancer gene expression data [27] are from The Cancer Genome Atlas (TCGA) [28]. All gene expression data were normalized by Z-score [29]. Biological process annotation terms which are used to construct gene annotation matrix are downloaded from Gene Ontology Consortium [30]. The performance of the proposed method is compared with the state-of-the-art methods [15]. Breast cancer gene expression data can be divided into multiple groups to optimize gene set annotation data according to breast cancer subtypes [31]. The enrichment power, enrichment replication and the area under ROC curve (AUC) were used to evaluate the proposed and other methods. The ROC curve was plotted based on the non-core genes as true positives and the genes filtered by the optimized annotation as predicted true positives. Here, genes were labeled with core or non-core using the GSEA algorithm and the designation of each gene set member by the GSEA algorithm as either a core gene or non-core gene with respect to enrichment against phenotypes of gene expression data was used as proxy for gene set annotation validation, that is, non-core genes mean that it’s not important for phenotypes of gene expression data, thus, we need to filter out genes (non-core) by the annotation optimization algorithm. In this way, we can construct some contingency table statistics to draw the ROC curves and calculate the AUC values to evaluate optimization algorithms.

#### Experiment on MSigDB C2 v1.0 gene sets and p53 gene expression data

The proposed method was performed on the curated MSigDB C2 v1.0 gene sets and the p53 gene expression data. With a minimum gene set size of 15 and maximum gene set size of 200, 301 of original 522 gene sets were used in our experiment. The results of optimization methods were generated on 50 bootstrap resampled datasets derived from the normalized p53 gene expression data. All genes were clustered by executing the k-means algorithm with the parameter k ranging from 3 to 15. The elements of final optimized annotation matrix were set to 0, if its value is less than 0.1 [15]. The performance evaluation of the annotation optimization method is difficult, in order to better evaluate the annotation results, we first used GSEA to determine the core genes and non-core genes of each gene set, thereby calculating the area under ROC curve [32] to evaluate algorithms. The improvement in enrichment power was quantified using the Benjamini’s false discovery rates (FDR) [33] calculated by the Correlation Adjusted Mean RAnk (CAMERA) [34] competitive enrichment method using the R implementation in the *limma* [35] package. The evaluation in enrichment replication was quantified using Kendall’s W(Kendall’s coefficient of concordance) [36] calculated on 20 bootstrap resample gene expression data. It was implemented by *irr* [37] package.

#### Experiment on MSigDB C4 v6.0 cancer modules and colon cancer gene expression data

The proposed method was performed on the MSigDB C4 v6.0 cancer modules [38] and colon cancer gene expression data. Similarly, 302 of original 431 cancer modules were used in our experiment. MSigDB cancer modules were generated by the analysis and integration of multiple data. Its specificity was not significant. Thus, the cancer modules should be optimized through gene expression data to increase its specificity. At the same time, differentially expressed genes have more representative significance in the expression data. MSigDB cancer modules were filtered by t-test to chase down differentially expressed genes. Similar to the parameter settings in the previous section, the results of optimization methods were generated on 50 bootstrap resampled datasets derived from the normalized colon cancer gene expression data. All genes were clustered by executing the k-means algorithm with the parameter *k* ranging from 3 to 15. The elements of final optimized annotation matrix were set to 0, if its value is less than 0.1 [15]. The CAMERA algorithm and Kendall’s coefficient of concordance were also used to evaluate the proposed and other methods.

#### Experiment on MSigDB C4 v6.0 cancer modules and breast cancer gene expression data

In this section, MSigDB C4 v6.0 cancer modules and breast cancer gene expression data were introduced into evaluate the optimization methods. For breast cancer gene expression data, we chose samples with positive estrogen receptor and four subtypes of Luminal A, Luminal B, HER2-enriched and Normal-like as experimental data. 310 of original 431 cancer modules were used in our experiment. Similar to the parameter settings in the previous section, MSigDB cancer modules were filtered by t-test to chase down differentially expressed genes. The relevant experimental parameters were consistent with the p53 gene expression data and colon cancer gene expression data.

### Experimental results

#### Results of MSigDB C2 v1.0 gene sets and p53 gene expression data

**Fig. 2:**
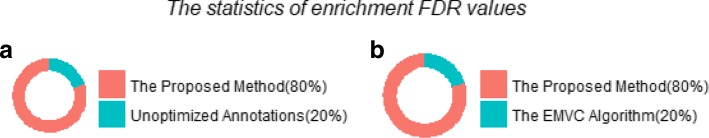
The enrichment FDR values calculated by unoptimized annotations, EMVC-optimized annotations and the-proposed-method-optimized annotations using p53 gene expression data. The ratio of different colors indicates the number of minimum FDR values compared to the two methods

The enrichment FDR values and *P*-values of all 301 MSigDB C2 v1.0 gene sets were calculated by unoptimized annotations, the proposed method and EMVC using CAMERA method. The results of all gene sets were sorted by the *P*-values of the unoptimized annotations and the top 15 gene sets were used to show evaluation results. Compared with unoptimized annotations, EMVC did not make any reduction in the enrichment FDR values for all 15 gene sets. The proposed method could effectively reduce the enrichment FDR values for 12 gene sets among 15 gene sets (80%) as shown in Fig. 2a. Compared with EMVC, the proposed method also reduced the enrichment FDR values for 12 gene sets (80%) as shown in Fig. 2b. The proposed method effectively improved the enrichment power on MSigDB C2 v1.0 and p53 gene expression data.

The area under ROC curve is calculated by genes which were designated as core or non-core by GSEA algorithm for gene sets. As shown in Table 1, Area under the ROC curve (AUC) calculated by the EMVC algorithm is 0.489. The AUC value calculated by the proposed method was 0.515. This demonstrated that the proposed method effectively removed the inconsistent annotations for p53 gene expression data.

**Table 1:** The Kendall’s W and AUC on MSigDB C2 v1.0 and p53 gene expression data

	The proposed method	The EMVC algorithm
Kendall’s W	0.325	0.322
AUC	0.515	0.489

The Kendall’s W values calculated by the unoptimized annotations, EMVC and the proposed method respectively (Table 1) were 0.323, 0.322 and 0.325. The unoptimized annotations and EMVC showed slightly differences on the enrichment replication (Kendall’s W). As shown in Table 1, the proposed method had better performance on the impact of enrichment replication than the EMVC algorithm and the unoptimized annotations.

#### Results of MSigDB C4 v6.0 cancer modules and colon cancer gene expression data

**Fig. 3:**
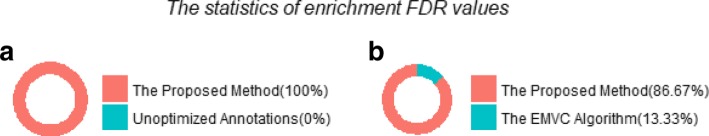
The enrichment FDR values calculated by unoptimized annotations, EMVC-optimized annotations and the-proposed-method-optimized annotations using colon cancer gene expression data. The ratio of different colors indicates the number of minimum FDR values compared to the two methods

In this section, we evaluated the optimization algorithms using MSigDB C4 v6.0 cancer modules and colon cancer gene expression data. The enrichment FDR values and *P*-values of all 302 MSigDB C4 v6.0 cancer modules were calculated by unoptimized annotations, EMVC and the proposed method using CAMERA method. The results of all gene sets were sorted by the *P*-values of the unoptimized annotations and the top 15 cancer modules were used to show evaluation results. The EMVC algorithm made the reduction to unoptimized annotations in enrichment FDR values for the top 15 MSigDB C4 v6.0 cancer modules. As shown in Fig. 3a, the proposed method made the reduction in enrichment FDR values for the top 15 cancer modules (100%). And the enrichment FDR values of 13 cancer modules (86.67%) calculated by the proposed method were lower than EMVC as shown in Fig. 3b. In summary, all of these demonstrated that the proposed method had better performance than EMVC on the impact of enrichment power.

As shown in Table 2, the AUC value calculated by the EMVC algorithm is 0.622. The AUC value calculated by the proposed method was 0.642. This demonstrated that the proposed method had better performance than EMVC in removing inconsistent annotations for colon cancer gene expression data.

**Table 2:** The Kendall’s W and AUC on MSigDB C4 v6.0 and colon cancer gene expression data

	The proposed method	The EMVC algorithm
Kendall’s W	0.975	0.975
AUC	0.642	0.622

The Kendall’s W calculated by the unoptimized annotations was 0.968. As shown in Table 2, The Kendall’s W calculated by EMVC and the proposed method were 0.975 and 0.975 respectively. The proposed method had a same performance on the impact of enrichment replication as the EMVC algorithm.

#### Results of MSigDB C4 v6.0 cancer modules and breast cancer gene expression data

**Fig. 4:**
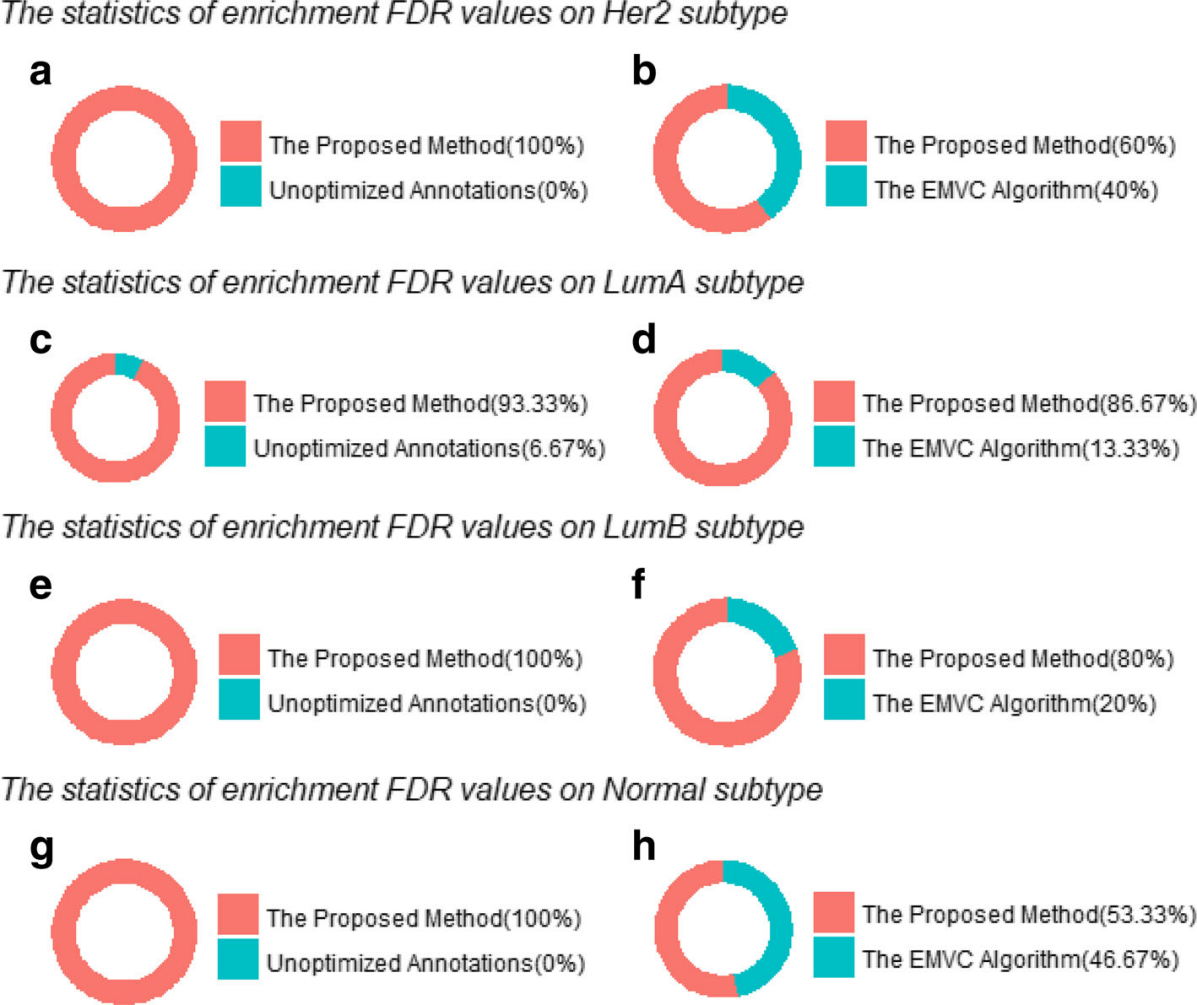
The enrichment FDR values calculated by unoptimized annotations, EMVC-optimized annotations and the-proposed-method-optimized annotations using breast cancer subtype gene expression data. The ratio of different colors indicates the number of minimum FDR values compared to the two methods

In this section, MSigDB C4 v6.0 cancer modules and breast cancer gene expression data were introduced to evaluate optimization algorithms. Breast cancer gene expression data samples were divided into four groups according to subtypes that were HER2-enriched, Luminal A, Luminal B and Normal-like. Analogously, the top 15 cancer modules were used to show evaluation results. The Fig. 4 intuitively showed comparison results of the enrichment FDR values for the top 15 cancer modules obtained by unoptimized annotations, the proposed method and EMVC using HER2-enriched, Luminal A, Luminal B and Normal-like gene expression data.

Compared with the unoptimized annotations, the EMVC algorithm made the reduction in enrichment FDR values for all of the 15 most significant cancer modules using HER2-enriched, Luminal A, Luminal B and Normal-like gene expression data respectively. The proposed method made the reduction to unoptimized annotations in enrichment FDR values for 15, 14, 15 and 15 among the 15 most significant cancer modules using HER2-enriched, Luminal A, Luminal B and Normal-like gene expression data respectively (Fig. 4a, c, e, g). Then EMVC and the proposed method were compared. The enrichment FDR values of 9, 13, 12 and 8 cancer modules (60%, 86.67%, 80%, 53.33%) calculated by the proposed method were lower than EMVC for HER2-enriched, Luminal A, Luminal B and Normal-like gene expression data respectively (Fig. 4b, d, f, h). Both of the proposed method and EMVC algorithm had an improvement to unoptimized annotations on enrichment power for breast cancer subtype gene expression data. However, the proposed method showed the best performance than EMVC on the impact of enrichment power.

Table 3 showed the AUC values calculated by the proposed method and the EMVC algorithm using different breast cancer subtype gene expression data. Especially for HER2-enriched and Luminal B breast cancer gene expression data, the proposed method was significantly better than the EMVC algorithm. This demonstrated that the proposed method effectively removed the inconsistent annotations on breast cancer gene expression data.

**Table 3:** The AUC on MSigDB C4 v6.0 and breast cancer subtype gene expression data

AUC	HER2-enriched subtype	Luminal A subtype	Luminal B subtype	Normal-like subtype
The proposed method	0.570	0.617	0.646.	0.563
The EMVC algorithm	0.540	0.614	0.635	0.562

As shown in Table 4, the proposed method and EMVC got better performance than unoptimized annotations on the enrichment replication (Kendall’s W). For HER2-enriched and Luminal B subtype, the performance of EMVC algorithm was slightly better than the proposed method. For Luminal A subtype, these two optimization algorithms had the same performance. For Normal-like subtype, the proposed method had better than EMVC. In summary, the performance of EMVC on the enrichment replication was slightly better than the proposed method using four subtype gene expression data, but it is not significant.

**Table 4:** The Kendall’s W on MSigDB C4 v6.0 and breast cancer subtype gene expression data

Kendall’s W	HER2-enriched subtype	Luminal A subtype	Luminal B subtype	Normal-like subtype
The proposed method	0.978	0.980	0.984	0.963
The EMVC algorithm	0.979	0.980	0.985	0.961
Unoptimized annotations	0.969	0.969	0.978	0.935

## Discussion

It is important to annotate gene functions for understanding the mechanism of complex diseases. However, the results of gene annotation based on the existing methods are relatively broad and not specific for different diseases. In the study, we optimize the results of gene set annotation by combining GO structure and gene expression data. Experimental results on several data shown that the proposed method improves the quality of gene set annotations, for example, the inconsistent annotations are filtered out, the enrichment power and the enrichment replication are effectively improved.

The proposed method is based on GO structure and gene expression data. The structure information of GO and gene annotations are still incomplete, in addition, there are noises in gene expression data due to experimental techniques and to the research bias in biology. Researchers from gene ontology and Microarray techniques are working hard to solve these problems. With the development of GO and microarray techniques, the proposed method may achieve better results.

## Conclusions

Gene set annotations play a pivotal role in the analysis and interpretation of genomic data. Although some gene set annotation methods have been developed, the results of gene set annotations are not special enough for different experimental data. In this article, we proposed a method to remove the inconsistency of gene set annotations and improve the quality of gene set annotations. Experiment results on the GO annotations of the genes and different gene expression data confirmed the effectiveness of the proposed method. Compared with the state-of-the-art methods, the proposed method improved effectively the enrichment power and remove the inconsistent of gene set annotations. For different gene expression data, the proposed method provides the optimized results of gene set annotations.

## Abbreviations

AUC: Area under the curve
DAG: Directed acyclic graph
DAVID: The database for annotation, visualization and integrated discovery
DE: Diferentially expressed
dRW: Downward random walk
EMVC: Entropy minimization over variable clusters
GO: Gene ontology
GOssTo: Gene ontology semantic similarity tool
GSEA: Gene set enrichment analysis
KEGG: Kyoto encyclopedia of genes and genomes
MSigDB: Molecular signatures database
pLSA: Probabilistic latent semantic analysis
ROC: Receiver operating characteristic curve
SVD: Singular value decomposition
TCGA: The cancer genome atlas

## References

[1] Subramanian A, Tamayo P, Mootha VK, Mukherjee S, Ebert BL, Gillette MA, Paulovich A, Pomeroy SL, Golub TR, Lander ES (2005). Gene set enrichment analysis: a knowledge-based approach for interpreting genome-wide expression profiles. Proc Natl Acad Sci.

[2] Mootha VK, Lindgren CM, Eriksson K-F, Subramanian A, Sihag S, Lehar J, Puigserver P, Carlsson E, Ridderstråle M, Laurila E (2003). Pgc-1 *α*-responsive genes involved in oxidative phosphorylation are coordinately downregulated in human diabetes. Nat Genet.

[3] Ogata H, Goto S, Sato K, Fujibuchi W, Bono H, Kanehisa M (1999). Kegg: Kyoto encyclopedia of genes and genomes. Nucleic Acids Res.

[4] Ashburner M, Ball CA, Blake JA, Botstein D, Butler H, Cherry JM, Davis AP, Dolinski K, Dwight SS, Eppig JT (2000). Gene ontology: tool for the unification of biology. Nat Genet.

[5] Khatri P, Sirota M, Butte AJ (2012). Ten years of pathway analysis: current approaches and outstanding challenges. PLoS Comput Biol.

[6] Masseroli M, Chicco D, Pinoli P. Probabilistic latent semantic analysis for prediction of gene ontology annotations. In: Neural Networks (IJCNN), The 2012 International Joint Conference on. IEEE: 2012. p. 1–8.

[7] Khatri P, Done B, Rao A, Done A, Draghici S (2005). A semantic analysis of the annotations of the human genome. Bioinformatics.

[8] Frasca M, Bertoni A, Re M, Valentini G (2013). A neural network algorithm for semi-supervised node label learning from unbalanced data. Neural Netw.

[9] Frasca M (2015). Automated gene function prediction through gene multifunctionality in biological networks. Neurocomputing.

[10] Yu G, Zhu H, Domeniconi C, Liu J (2015). Predicting protein function via downward random walks on a gene ontology. BMC Bioinformatics.

[11] Huang DW, Sherman BT, Lempicki RA (2008). Systematic and integrative analysis of large gene lists using david bioinformatics resources. Nat Protoc.

[12] Huang DW, Sherman BT, Lempicki RA (2008). Bioinformatics enrichment tools: paths toward the comprehensive functional analysis of large gene lists. Nucleic Acids Res.

[13] Faria D, Schlicker A, Pesquita C, Bastos H, Ferreira AE, Albrecht M, Falcão AO (2012). Mining go annotations for improving annotation consistency. PLoS ONE.

[14] Binns D, Dimmer E, Huntley R, Barrell D, O’donovan C, Apweiler R (2009). Quickgo: a web-based tool for gene ontology searching. Bioinformatics.

[15] Frost HR, Moore JH (2014). Optimization of gene set annotations via entropy minimization over variable clusters (emvc). Bioinformatics.

[16] Tong H, Faloutsos C, Pan J-Y (2008). Random walk with restart: fast solutions and applications. Knowl Inf Syst.

[17] Valentini G (2011). True path rule hierarchical ensembles for genome-wide gene function prediction. IEEE/ACM Trans Comput Biol Bioinforma (TCBB).

[18] Caniza H, Romero AE, Heron S, Yang H, Devoto A, Frasca M, Mesiti M, Valentini G, Paccanaro A (2014). Gossto: a stand-alone application and a web tool for calculating semantic similarities on the gene ontology. Bioinformatics.

[19] Lin D (1998). An information-theoretic definition of similarity. Citeseer.

[20] Liberzon A, Subramanian A, Pinchback R, Thorvaldsdóttir H, Tamayo P, Mesirov JP (2011). Molecular signatures database (msigdb) 3.0. Bioinformatics.

[21] Liberzon A, Birger C, Thorvaldsdóttir H, Ghandi M, Mesirov JP, Tamayo P (2015). The molecular signatures database hallmark gene set collection. Cell Syst.

[22] Patkar S, Magen A, Sharan R, Hannenhalli S (2017). A network diffusion approach to inferring sample-specific function reveals functional changes associated with breast cancer. PLoS Comput Biol.

[23] Zhang K, Cui S, Chang S, Zhang L, Wang J (2010). i-gsea4gwas: a web server for identification of pathways/gene sets associated with traits by applying an improved gene set enrichment analysis to genome-wide association study. Nucleic Acids Res.

[24] Delmore JE, Issa GC, Lemieux ME, Rahl PB, Shi J, Jacobs HM, Kastritis E, Gilpatrick T, Paranal RM, Qi J (2011). Bet bromodomain inhibition as a therapeutic strategy to target c-myc. Cell.

[25] Zhang B, Wang J, Wang X, Zhu J, Liu Q, Shi Z, Chambers MC, Zimmerman LJ, Shaddox KF, Kim S (2014). Proteogenomic characterization of human colon and rectal cancer. Nature.

[26] P, 53 Cell Lines. http://www.gsea-msigdb.org/gsea/datasets.jsp. Accessed 1 Jan 2018.

[27] TCGA Legacy Archive. https://portal.gdc.cancer.gov/legacy-archive/search/f. Accessed 1 Jan 2018.

[28] Tomczak K, Czerwińska P, Wiznerowicz M (2015). The cancer genome atlas (tcga): an immeasurable source of knowledge. Contemp Oncol.

[29] Altman EI (1968). Financial ratios, discriminant analysis and the prediction of corporate bankruptcy. J Financ.

[30] Consortium TGO (2015). Gene ontology consortium: going forward. Nucleic Acids Res.

[31] Sørlie T, Perou CM, Tibshirani R, Aas T, Geisler S, Johnsen H, Hastie T, Eisen MB, Rijn MVD, Jeffrey SS (2001). Gene expression patterns of breast carcinomas distinguish tumor subclasses with clinical implications. Proc Natl Acad Sci.

[32] Green DM, Swets JA. Signal detection theory and psychophysics. 1966;1478–1481.

[33] Benjamini Y, Hochberg Y (1995). Controlling the false discovery rate: A practical and powerful approach to multiple testing. J R Stat Soc.

[34] Di W, Smyth GK (2012). Camera: a competitive gene set test accounting for inter-gene correlation. Nucleic Acids Res.

[35] Smyth GK. limma: Linear Models for Microarray Data. Bioinform Comput Biol Solutions Using R Bioconductor. 2011;:397–420.

[36] Kendall MG, Smith BB. The problem of m rankings: 1939. p. 275–87.

[37] irr: Various Coefficients of Interrater Reliability and Agreement. https://cran.r-project.org/web/packages/irr/index.html.

[38] Segal E, Friedman N, Koller D, Regev A (2004). A module map showing conditional activity of expression modules in cancer. Nat Genet.

